# The role of general practitioners in the work guidance of cancer patients: views of general practitioners and occupational physicians

**DOI:** 10.1007/s11764-022-01211-1

**Published:** 2022-04-25

**Authors:** Marie-Christine Sarfo, Lucinda Bertels, Monique H. W. Frings-Dresen, Femke de Jong, Annette H. Blankenstein, Kristel M. van Asselt, Angela G. E. M. de Boer

**Affiliations:** 1 Department of Public and Occupational Health, Coronel Institute of Occupational Health, Amsterdam Public Health Research Institute, Amsterdam University Medical Centers, University of Amsterdam, Meibergdreef 9 / K0-119, 1105 AZ Amsterdam, The Netherlands; 2 Department of General Practice/Family Medicine, APH, Amsterdam University Medical Centers, University of Amsterdam, Amsterdam, The Netherlands; 3 Department of Socio-Medical Sciences, Erasmus School of Health Policy & Management, Erasmus University Rotterdam, Rotterdam, The Netherlands; 4 Department of General Practice/Family Medicine, Amsterdam University Medical Centers, Vrije Universiteit, Amsterdam, The Netherlands

**Keywords:** General practitioner, Occupational physician, Return to work, Cancer survivors

## Abstract

**Purpose:**

To explore views of general practitioners (GPs) and occupational physicians (OPs) on the role of GPs in work guidance of cancer patients.

**Methods:**

Between 2016 and 2019, two focus groups with GPs (*N* = 17) and two focus groups with OPs (*N* = 10) were conducted. Focus group discussions were audiotaped and transcribed verbatim. Transcripts were analysed by data-driven analysis.

**Results:**

GPs generally indicated that they inquire about patients’ occupations but do not structurally document these. GPs described offering support and advice to patients regarding their work, while other GPs stated they do not interfere with their patients’ work or return to work (RTW) process.

In general, GPs stated that they do not aspire a professional role in the work guidance of patients, due to lack of expertise and not having sufficient knowledge in work regulations and legislation. In contrast, OPs anticipated a proactive role from GPs concerning work guidance in cancer patients, and they expected GPs to refer cancer patients to the OP, when required. Moreover, they emphasised the importance of communication between GPs and OPs about patients’ work-related problems to achieve common goals.

**Conclusions:**

GPs can contribute to cancer patients’ RTW process by supporting patients, giving advice and providing referral to other health professionals. Better cooperation between GPs and OPs may improve work guidance in cancer patients.

**Implications for Cancer Survivors:**

When cancer patients with work-related issues get appropriate advice and support from GPs and referred in time to OPs, the RTW process and staying at work of cancer patients may be positively affected.

## Introduction

The incidence of cancer diagnoses in working-age patients has increased over the years. Worldwide, 19.3 million new cancer cases were estimated to have occurred in 2020 [1]. Due to cancer diagnosis and treatment with e.g. surgery and chemotherapy, cancer patients often temporarily have to stop working [2]. During cancer diagnosis, and before and after treatment, cancer patients can experience work-related problems that lead to absence from work. Unfortunately, some of these patients do not return to work at all [3]. However, many cancer patients are motivated to return to work [4] because they value work and believe that it is one of the most important aspects of their life [5].

Although many cancer patients can work after treatment, research has shown that their work ability is poorer compared to healthy controls [6–10]. Furthermore, cancer patients are at higher risk of job loss and early retirement [11]. However, returning to work can be beneficial and even necessary for cancer patients to prevent, for example, financial problems [12, 13].

In the Netherlands, occupational physicians (OPs) are employed by companies and can provide specialist, work-based guidance. They have a coaching role regarding cancer patients, with the aim of continued employment and return to work. The interventions of OPs focus on the recovery of the work function of cancer patients.

Psychological support and consultation with a physician during sick leave can have a positive effect on the return to work (RTW) rate [14]. Previous studies suggest that healthcare professionals, such as general practitioners (GPs), could aid cancer patients by providing general work guidance [4, 15–17] which includes general advice about work ability and return to work. However, the current general guidance related to work that cancer patients receive from healthcare professionals is limited [15]. The limitations to provide work guidance include lack of possibilities to assess work readiness and to identify supports available in the workplace [15].

Although the importance of work guidance by healthcare professionals for cancer patients has been acknowledged, little is known about GPs’ perspectives on their perceived professional role in the work guidance of these patients. Furthermore, little is known about the views of occupational physicians (OPs) on the role of GPs in the work guidance of cancer patients.

The aim of this study is therefore to explore the perspectives of GPs and OPs regarding the professional role of GPs in the work guidance of cancer patients.

The following research questions are addressed in the present article: (1) Do general practitioners inquire about and document the professions of their patients’ professions? (2) What role do general practitioners perform in the work guidance and return to work of cancer patients? (3) How do general practitioners consider their professional role in the work guidance and return to work of cancer patients? (4) What role do occupational physicians expect general practitioners to have in the work guidance and return to work of cancer patients? and (5) How do general practitioners and occupational physicians communicate with each other about work guidance and return to work of cancer patients?

## Methods

The Consolidated Criteria for Reporting Qualitative Research (COREQ) checklist was used to report the methods and results of this qualitative study [18]. Ethical approval was waived by the Medical Ethical Committee of the Amsterdam UMC, as The Medical Research Involving Human Subjects Act (WMO) does not apply to this study (W16_140 # 16.163).

### Participant recruitment and setting

In November 2016, September 2017, and November 2019, an experienced moderator (KvA) and two trained observers (FdJ and MCS) conducted four focus groups. These four focus groups consisted of two groups with GP participants (2016) and two groups with OP participants (2017; 2019). GPs received an e-mail in which they could sign up to participate in focus groups about the psychosocial effects of breast cancer and the role of the GP. The GPs who participated received information about the focus group before taking part. All GPs were teaching physicians, guiding general practitioners in training. The participants of the two OP focus groups were pre-existing intercollegiate consultation groups and selected by convenience sampling and they were recruited by e-mail via two OPs. Participants were further informed about the content of the focus group by researchers (FdJ, MCS). All OP participants received an e-mail notification letter prior to the focus group and were aware and well informed about the content of the focus group. A total of 27 health providers, featuring 17 GPs and 10 OPs, participated in the study.

There was no relationship established between the researchers and any of the focus groups participants prior to the study. During the focus groups, the moderator and the observer introduced themselves. They shared their function and the reasons for conducting the investigation, namely, their interest in the experiences and opinions of the participants on the research topics.

The data of the GP focus groups were collected in a conference centre and the data of the two OP focus groups were obtained at the houses of two different OPs. Participants completed a short questionnaire to assess demographics, including age, years of experience, and working hours per week (Table 1). All participants provided their informed consent in writing before participating.

**Table 1:** Demographics of the general practitioners (*n* = 17) and occupational physicians (*n* = 10)

	General practitioners	Occupational physicians
	*n* (%)	*n* (%)
Gender		
Male Female	8 (47.1%) 9 (52.9%)	6 (60%) 4 (40%)
Age (years)	Range: 41–61 Mean: 53.1 SD: 7.4	Range: 41–62 Mean: 52.2 SD: 7.7
Years of experience	Range: 10–32 Mean: 20.5 SD: 7.7	Range: 14–30 Mean: 20 SD: 5.8
Working days per week	Range: 3–5 Mean: 3.7 SD: 0.8	Range: 2–5 Mean 3.9 SD: 0.9
Working region		
City Village	13 (76.5%) 4 (23.5%)	Not applicable

*SD*, standard deviation

### Data collection

The focus groups were guided based on topic guide books (Table 2). The focus group data were audio-recorded. During the focus groups, field notes were made by the observers (FdJ, MCS). The mean duration of the four focus groups was 63 min.

**Table 2:** GP/OP focus groups topic guides

GP focus group
Actively asking about work by the GP The moment of first contact between the GP and thecancer patient The way in which contact is made between the GP and the cancer patient The initiative taken for contact from the GP or cancer patient The rules in the standard care appointments about contact with cancer patients The current role of the GP in cancer care regarding work or reintegration The GP’s perspectives to undertake the work reintegration of cancer patients The steps required for the GP to pay attention to the work reintegration of cancer patients The moment when the GP should start talking about work with cancer patients The opportunities for the GP’s role regarding cancer and work or reintegration
OP focus group
The role of the occupational physician regarding cancer patients and return to work The collaboration or communication between the OP and GP regarding cancer patients and their work reintegration The contact between OP and GP regarding cancer patients The timing of the contact between OPs and GPs The way in which contact takes place between OPs and GPs The content of the contact between OPs and GPs (explicit discussion of return to work or medical content (diagnosis/medical history)) The specific work-related issues that OPs do not discuss with the cancer patient but leave to the GP The opinion of OPs about the current role of the GP regarding cancer patients and the consequences for work/return to work The perspective of OPs on the work reintegration of cancer patients as a task for the GP (for every cancer patient or a specific target group, e.g. self-employed) The OPs perspectives on the ideal role of the GP in the work reintegration of cancer patients The perspectives of the OPs on the way in which the GP should respond to questions about (return to) work The OPs perspectives on actively asking about work issues of cancer patients by the GP The coordination between the tasks of the OPs and GPs The OPs perspectives on when the GP should start asking about work with cancer patients The perspectives of the OPs on the role of the GP with regard to work issues (in relation to the relationship of trust and the distance from work/the neutral position as a GP with regard to work) The care for the self-employed who do not have a company doctor or OP

### Data analysis

The focus group data was transcribed. Transcripts were read at least two times and initial ideas were noted by the two coders LB and MCS.

Open coding was performed by using the analysis programme MAXQDA 2020 [19]. LB and MCS independently identified meaningful units and labelled them with a code. LB independently open-coded the first GP focus group and the first OP focus group. MCS independently open-coded all four focus groups. The codes were generated inductively, and a data-driven approach was used. The coders discussed their initial codes and findings with each other. A coding tree explaining the meaning of the codes was created and discussed with the research team (MCS, LB, MF, AB, AdB). Codes were grouped into themes by the two coders and again discussed with the research team. The themes were defined and named; all adjustments were discussed with the research team until consensus was reached. The most relevant quotes were selected and discussed.

## Results

In total, 17 GPs participated in two GP focus groups and 10 OPs participated in two OP focus groups (Table 1). The obtained results are presented below per research question. Table 3 represents illustrative quotes from the four focus groups.

**Table 3:** Illustrative quotes about GPs and OPs views on the role of GPs in work guidance and return to work of cancer patients

Themes	Subthemes	Quotes
Inquire and document patients’ professions	Inquire about patients’ professions	‘I really do focus on work. I also ask it during introductory meetings, and I also like to ask further questions. Because on the basis of someone’s work, you often get to know someone’s character a little bit better.’ (GP, P3) ‘Whenever I think in some way that it (work) can have an, I will ask.’ (GP, P8) ‘Work is important, but yes, I find it very difficult to always include it.’ (GP, P7)
	Registration of patient’s professions in GP information system	‘I think I almost always ask about work (uhhm) but I don’t put it, say, as a problem… It’s not structurally documented somewhere.’ (GP, P4)
GPs perspectives of their role in work guidance and return to work of cancer patients	Advice and supportive role	‘I have the idea that you don’t see the people who are doing well. You have to slow half of them down and stimulate the other half.’ (GP, P17) ‘I often ask: can you do this, can you do this whole thing; how, how does your employer react to it? I ask: do you get the opportunity to be sick?’ (GP, P15)
	Referral role	‘Well, people get stuck in their work just like with burnout. Well, I think that is also very suitable to initially go to the mental health care nurse.’ (GP, P14) ‘I never refer to the OP, that is always through the employer.’ (GP, P15)
	Work guidance and return to work is not a GP’s job	‘I don’t think it’s really my expertise to guide people with cancer to back to work.’ (GP, P17) ‘I don’t think it’s up to me to judge if they are allowed to work.’ (GP, P10) ‘You can really get into a conflict situation if you, as a GP, are given a very important role in it. I think we should really stay out of that.’ (GP, P9) ‘I only see a role for myself in encouraging someone to go to work in the interest of that patient.’ (GP, P15)
The role OPs expect from GPs in continuing employment and work reintegration of cancer patients	Proactive role	‘A more proactive role from the GP and also from the medical specialist with regard to work.’ (OP, P7) ‘What I expect from a GP is that he actually has, in principle, a willingness to consult with us as colleagues. And that is not the case with all GPs. There is still a strong distrust.’ (OP, P2) ‘What I expect from the GP is that the GP also discusses work issues and also indicates what the added value of work could be, or refer to the OP.’ (OP,P5)
	Educate GPs about OPs’ role in continued employment and return to work	‘It would help enormously if the GPs were well aware of our role and what our possibilities are. Because you also see that GPs often have the impression that the only thing we care about is sending people back to work.’ (OP,P3)
The communication between GPs and OPs about the work guidance and return to work of cancer patients	GPs perspectives about communication with OPs	‘What I do think: contact with the OP is very scarce. Very little. Yes, I think it is a pity.’ (GP, P7) ‘I think the OP and I should strive for the same thing. Even when there is a conflict.’ (GP, P12)
	OPs perspectives about communication with GPs	‘It is actually quite rare for the GP to make contact himself. That’s all fine. The other way around it is just the same.’ (OP, P10) ‘Nevertheless, it makes sense to confer, because you are on the same page together and you create that by conferring with the GP.’ (OP, P1)

### Research question 1: Do general practitioners inquire about and document their patients’ professions?

#### Inquiring about patients’ professions

GPs stated they often enquired about patients’ occupations, usually when new patients register in their practice. Some GPs described that they find it important to be aware of these occupations. A GP mentioned that it provides him with the opportunity to get to know the personality of a patient. Although work is considered important to patients, GPs mentioned it is sometimes inconvenient to discuss work with patients during a consultation.

#### Registration of patients’ professions in the GP information system

GPs in the Netherlands utilize about 10 different GP information systems which are software applications in which GPs document data of patients. Some GPs mentioned entering the professions of patients in the GP information system, but not all do so structurally.

Some information systems have a special section in which the professions of patients can be written down. Despite this, in practice, many of these sections are not filled out by GPs.

GPs stated that it will be more evident to involve the professions of patients if a role is assigned to the GP in cancer aftercare.

### Research question 2: What role do general practitioners play in work guidance and return to work of cancer patients?

#### Advice and supportive role

During the cancer trajectory of their patients, several GPs mentioned discussing psychosocial issues, including work. There are GPs who feel comfortable enough to give patients advice about work-related problems. They believe that this can be beneficial for the patient’s well-being. Furthermore, GPs discussed that patients have different coping styles concerning RTW and maintaining employment. According to GPs’ experience, some patients need to be stimulated to RTW, while others need to be advised to slow down and work less.

In the work guidance of patients, GPs mentioned they believe it is important to listen carefully to patients’ work-related problems. They stated that this often helps patients to come up with the answers to their own questions. One GP mentioned that some patients need a little push and a boost of confidence when struggling with work-related problems.

#### Referral role

Some GPs described that they refer patients to their primary care mental health care professional, in case of psychosocial problems and work-related problems. The primary care mental health care professional usually has more time to discuss these issues.

One GP mentioned not to refer patients directly to the OP, because referrals to OPs are mostly done by patients’ employers. Instead, most GPs stated that they advise patients to make an appointment with the OP. However, according to the GPs, the employers of patients do not always give patients permission to make such an appointment. GPs mentioned that they think that financial cost for the companies is one of the reasons why employers do not allow their employees to consult an OP.

### Research question 3: How do general practitioners consider their professional role in work guidance and the return to work of cancer patients?

#### Work guidance and return to work are not a GP’s task

One GP described seeing little difference between cancer patients and other patients with serious illness or chronic disease with respect to work guidance. Furthermore, the GP mentioned experiencing friction with her own standards and values regarding the time at which a patient should resume working. Some GPs stated that cancer patients currently receive enough guidance from health providers in the hospital and they do not consider work guidance or RTW to be their task. Furthermore, most GPs described they do not wish to get involved with patients’ legal rights to illness benefits. This is due to not having sufficient knowledge or experience in work regulations and legislation associated with these benefits and therefore feeling at risk of being involved in a conflict situation between employers and patients. As such, GPs described being hesitant with offering work guidance in the form of a direct recommendation to return to work. Rather, they stated that the importance of work and advice regarding work is discussed in the context of restoring patients’ overall well-being and balance, and generally as a part of aftercare and psychosocial guidance.

#### Notification from oncology specialists

GPs said that it is not always clear to them when one of their patients has finished their cancer treatment. GPs stated that although they receive letters from oncology specialists about the cancer trajectory, it is not clear at what point GPs are intended to be involved in cancer aftercare. GPs mentioned that it would be helpful to establish both their own role and that of the oncological specialist more clearly, including who should be tasked with work guidance and at what moment.

### Research Question 4: Which role do occupational physicians expect general practitioners to have in work guidance and return to work of cancer patients?

#### GPs and a proactive role in work guidance and return to work

In focus groups with OPs, they stated that they expect GPs to address work-related issues and inform patients about the added value of working. Furthermore, several OPs expected that GPs refer patients to them, when necessary. OPs described that there is a shared goal between GPs and OPs in improving patients’ return to work. As such, they mentioned that they expect GPs to be open to a discussion with them about work-related issues of patients. However, OPs described sensing distrust among GPs regarding the role of OPs. They said that it may be helpful to better inform GPs about their function as they feel that this distrust may not be justified.

### 3.5 Research Question 5: How do general practitioners and occupational physicians communicate with each other about work guidance and return to work for cancer patients?

#### Communication between GPs and OPs

GPs mentioned that, in general, contact between them and OPs about patient work-related problems is scarce. Some GPs explained that it can be difficult to get in contact with OPs when needed. One GP mentioned that he is not willing to contact the OP anymore, rather the OP should initiate contact. To provide work guidance, GPs believe they need to be aligned with OPs, or at least have the opportunity to discuss with OPs.

OPs mentioned that contact with GPs about cancer patients is very limited, and it mainly concerns requests of a patient’s medical information or in problematic situations. Conversely, several OPs said that GPs rarely contact them about work-related issues in cancer patients.

One OP stated that he involves a GP as much as possible during a cancer patient’s treatment. This OP believed that it is important for GPs to stay informed about their patients.

The opinions of the OPs on the reachability of GPs are divided. One OP shared that GPs are always easily accessible to communicate with. On the contrary, some OPs mentioned that GPs are difficult to reach. Furthermore, an OP shared that not all GPs are willing to consult with OPs, and there seems to be a strong mistrust among GPs towards OPs. Nevertheless, OPs wish to be informed about patients by GPs; they believe this can help with the work guidance of patients. OPs think it is useful to consult GPs to check if they are aligned.

## Discussion

Our study shows that GPs generally ask patients about their occupations, and that they sometimes document the occupation in a general practitioner information system. GPs give advice on work-related problems, and they sometimes refer cancer patients to their primary care mental health care professional to have further discussions on work-related issues. On the other hand, some GPs do not view work guidance and RTW of cancer patients as their domain, unless patients actively ask help for work-related issues. They lack expertise and want to avoid a conflict situation with the employer of their patients. Nonetheless, OPs expect a proactive role from GPs in work guidance of cancer patients. Both GPs and OPs mentioned that it can be difficult to get in contact with each other. However, both consider it important to have the opportunity to consult with each other about their cancer patients.

### Comparison with the existing literature

In France, Lamort-Bouché et al. (2020) studied the perspective of breast cancer specialists on their role in their patients RTW [20]. In this study, health providers had varying roles, ranging from non-involvement to frequent discussions with their patients. Compared to our study the barriers to involvement in patients RTW reported in their study were similar to ours. In our study, some GPs also mentioned that there is a lack of knowledge of work-related problems guidance, and that they mainly want to focus on the cancer care. Moreover, they do not want to interfere in the work guidance and RTW of cancer patients because they do not consider it their job due to not having sufficient knowledge or experience in work regulations and legislation. GPs in our study are willing to provide work guidance only if it benefits the welfare and recovery of their patients. Furthermore, an integrative, multidisciplinary approach towards health care and patient counselling is not always commonplace and health care providers might still be quite segregated in terms of specialisms. Hence, awareness of work-related problems could be raised in health care providers through trainings.

Similar to our study, health professionals in the study of Yagil et al. in Israel were confident in the benefits of continuing work and RTW in cancer patients [16]. In addition, health care professionals, including family practitioners, in their study view RTW as part of their responsibility as a caregiver. On the other hand, in our study, most GPs are unwilling to play a professional role in the work guidance and the RTW process of their cancer patients. In a focus group study from Germany with GPs and OPs and medical specialists, it was also concluded that OPs considered optimisation of cooperation necessary, while its necessity was sometimes questioned in the GPs' group [21]. The difference in opinion could be explained by the fact that in some countries providing sick leave certificates belongs to the task of GPs [21] or some GPs have additional qualifications or specialist training in occupational medicine [21]. However, in the Netherlands, it is the professional responsibility of the OP to handle and guide patients' work-related issues [22]. As a result, GPs and OPs in different countries might have different views on their perceived role in work guidance, depending on their country’s legislation.

### Strengths and limitations

This study identified the current status of GPs’ work guidance in cancer patients. To our knowledge, this is the first study that explored the perspectives of both GPs and OPs on the role of GPs in work guidance and return to work of cancer patients. By including both professions, it was possible to retrieve information on how GPs and OPs interact and how their collaboration could be improved.

A limitation of our study is the selection of GP and OP participants. We do not have information on the GPs and OPs who decided not to participate. It is possible that we have selected participants who would have a more than average positive or negative point of view on providing work guidance to cancer patients. A second limitation is that our study took place in 2016, 2017, and 2019 before the COVID-19 pandemic. GPs and OPs might have collaborated more and discussed the impact of return to work of vulnerable cancer patients during the pandemic. Otherwise, we do not expect any changes that would have impacted our data. Furthermore, data saturation may not have been achieved because new themes emerged when analysing the final focus groups. Therefore, it is possible that our data are not exhaustive.

### Implications for research and practice

Improvement in the registration of patient occupations in the GPs’ registration systems may benefit GPs’ abilities to assess the appropriateness of RTW of their patients. In addition, expanded communication from the oncological specialist regarding when a patient’s treatment has finished and improved communication between GPs and OPs could enhance the general work guidance of GPs for cancer patients. As motivational issues may be underlying, it may also be beneficial to investigate the potential of incentives for GPs who have an interest in providing RTW.

Studies have found that collaboration between health professionals has a positive effect on patient work guidance [21, 23]. Although in other countries, cooperation between GPs and OPs is also lacking or suboptimal, studies have reported that both GPs and OPs show interest in cooperation [21, 24]. Therefore, future research should focus on how to improve communication between health professionals, GPs, OPs, and oncologists, because this can help clarify the exact roles of health providers in the work guidance of cancer patients.

The Dutch College of General Practitioners (NHG) reported that GPs need to focus on the (return to) work of cancer patients during the cancer treatment phase [25]. In addition, the NHG breast cancer guideline describes various possibilities, in which GPs can support cancer patients to return to work [26]. Nevertheless, in our study, most GPs are not prepared to have a professional role in work guidance of cancer patients. Although improvement can be made in the guidelines for GPs, first awareness about the currently inadequate work guidance of cancer patients is needed in GPs and what their guidance could mean for the return to work of cancer patients. In the study of Kock et al., unfortunately, a 5-h training for physicians did not improve the registration of GPs for work-related problems and occupation [26]. The authors recommend the development of an intervention aimed at high-risk patients, tailored to the individual needs of the GP, and providing ongoing feedback [27].

The results we found regarding the role of the GP in work guidance, and possible cooperation between GPs and OPs, might not be specific to cancer patients. It is therefore quite possible that the results are generalizable to other chronic diseases. The first steps in strengthening the role of GPs in general work guidance of cancer patients, and possibly other workers with a chronic disease, could therefore be to enquire about the work situation and work ability of patients. If indicated, GPs can subsequently refer patients to OPs for specialist work guidance.

## Conclusions

GPs generally ask patients about their professions, and in some cases, they can document the occupation in an information system. GPs give advice on work-related problems and/or refer cancer patients to other healthcare professionals although some GPs do not view work guidance and RTW of cancer patients as their domain, unless patients actively ask for help for work-related problems. On the other hand, OPs expect a proactive role from GPs in work guidance of cancer patients. It may be beneficial to investigate the potential of incentives, education, or training for GPs who have an interest in providing RTW advice, to enhance motivation to provide work guidance. Both GPs and OPs mentioned that communication between the two professions can be difficult but should be stimulated because this could improve the work guidance in cancer patients.
